# Biomarkers in abnormal uterine bleeding^†^

**DOI:** 10.1093/biolre/ioy231

**Published:** 2018-11-01

**Authors:** Rohan Chodankar, Hilary O D Critchley

**Affiliations:** MRC Centre for Reproductive Health, Queen's Medical Research Institute, The University of Edinburgh, Edinburgh, UK

**Keywords:** angiogenesis, apoptosis, endometrium, estradiol/estradiol receptor, epigenetics, female reproductive tract, menstrual cycle, proteomics, progesterone/progesterone receptor, uterus

## Abstract

Abnormal uterine bleeding (AUB) is an extremely common problem and represents a clinical area of unmet need. It has clinical implications and a high cost for the healthcare system. The PALM-COEIN acronym proposed by FIGO may be used as a foundation of care; it improves the understanding of the causes of AUB, and in doing so facilitates effective history taking, examination, investigations, and management.

Heavy menstrual bleeding, a subset of AUB, is a subjective diagnosis and should be managed in the context of improving the woman's quality of life. Available evidence suggests that there is poor satisfaction with standard treatment options often resulting in women opting for major surgery such as hysterectomy. Such women would benefit from a tailored approach, both for diagnosis and treatment, highlighting the deficiency of biomarkers in this area.

This article focuses on the causes of AUB as per the PALM-COEIN acronym, the researched biomarkers in this area, and the potential pathogenetic mechanisms. In the future, these approaches may improve our understanding of AUB, thereby enabling us to direct women to most suitable current treatments and tailor investigative and treatment strategies to ensure best outcomes, in keeping with the principles of personalized or precision medicine.

## Teaser

Abnormal uterine bleeding (AUB) is an extremely common problem and represents a clinical area of unmet need. Identifying potential biomarkers in this area would allow for planning individualized care and improving treatment outcomes.

## Introduction

A biomarker is a characteristic that can be objectively measured and evaluated as an indicator of normal biological processes, pathogenic processes, or pharmacological responses to a therapeutic intervention. The ideal platforms for biomarker discovery include genomic, transcriptomic, proteomic, metabolomic, and imaging analyses. Many commonly used tests in clinical practice can serve as biomarkers, and the majority have been identified on the basis of insight or underlying physiology or biological mechanisms [1]. Abnormal uterine bleeding (AUB) is an extremely common problem and represents a clinical area of unmet need. Identifying potential biomarkers in this area would allow for planning individualized care.

AUB was re-defined by Federation International de Gynecologie et d’Obstetrique (FIGO) in 2009 to introduce standardization of nomenclature and identify an etiological basis [2, 3]. Chronic AUB was defined as bleeding from the uterine corpus that is abnormal in volume, regularity, and or timing and has been present for the majority of the last 6 months. Acute AUB was defined as an episode of heavy bleeding that, in the opinion of the clinician, is of sufficient quantity to require immediate intervention to prevent further blood loss. Intermenstrual bleeding is defined as bleeding that occurs between clearly defined cyclic and predictable menses and includes both randomly occurring episodes and those that manifest predictably at the same time in each cycle [2].

Heavy menstrual bleeding (HMB) is a subcategory of AUB and has a woman-centered approach to diagnosis. Rather than using objective measurements of volume or using PBAC (Pictorial Blood Assessment Chart) scores, NICE (National Institute for Care and Excellence) define HMB as an excessive menstrual loss that interferes with the physical, social, emotional, and or material quality of life [4]. This takes precedence over the previously used definitions of menstrual blood loss of greater than 80 mL in both research and clinical settings [5–7].

### History of menstrual terminology

From 430 BC until the early 1800s, the main menstrual symptom appearing in medical writings was excessively heavy bleeding [8]. The description of periods in early writings also includes phrases such as “the flux is immoderate, either when the periods return too often, when they continue too long, or when too much blood is discharged at one time.” Irregular and often light bleeding was referred to as “the weeping of the womb” [9].

The term “menorrhagia” appears to have been used for the first time in the late 1700s in the lectures of Professor William Cullen, Professor of the Practice of Physic at the University of Edinburgh. The term “metrorrhagia” probably came into use at the same time, with Cullen using the spelling “maetrorhagia” [10].

The introduction of the confusing modern term “dysfunctional uterine bleeding” did not occur until the 1930s. Graves used the term “dysfunctional uterine bleeding” to try and explain “impairment of endocrine factors,” which normally controlled menstrual function [11].

### FIGO classification of AUB etiology

Once a diagnosis of AUB has been established, the further classification is based on the PALM-COEIN acronym. The system was developed with contributions from an international group of both clinical and nonclinical investigators from 17 countries on six continents [3]. This system was created as a detailed assessment of the previously used menstrual terminology concluded that there is great confusion in the way these terminologies are used and there is an urgent need for international agreement on the consistent use of terms and definitions for symptoms, signs, and causes of abnormal uterine bleeding [12, 13].

**Figure 1.**
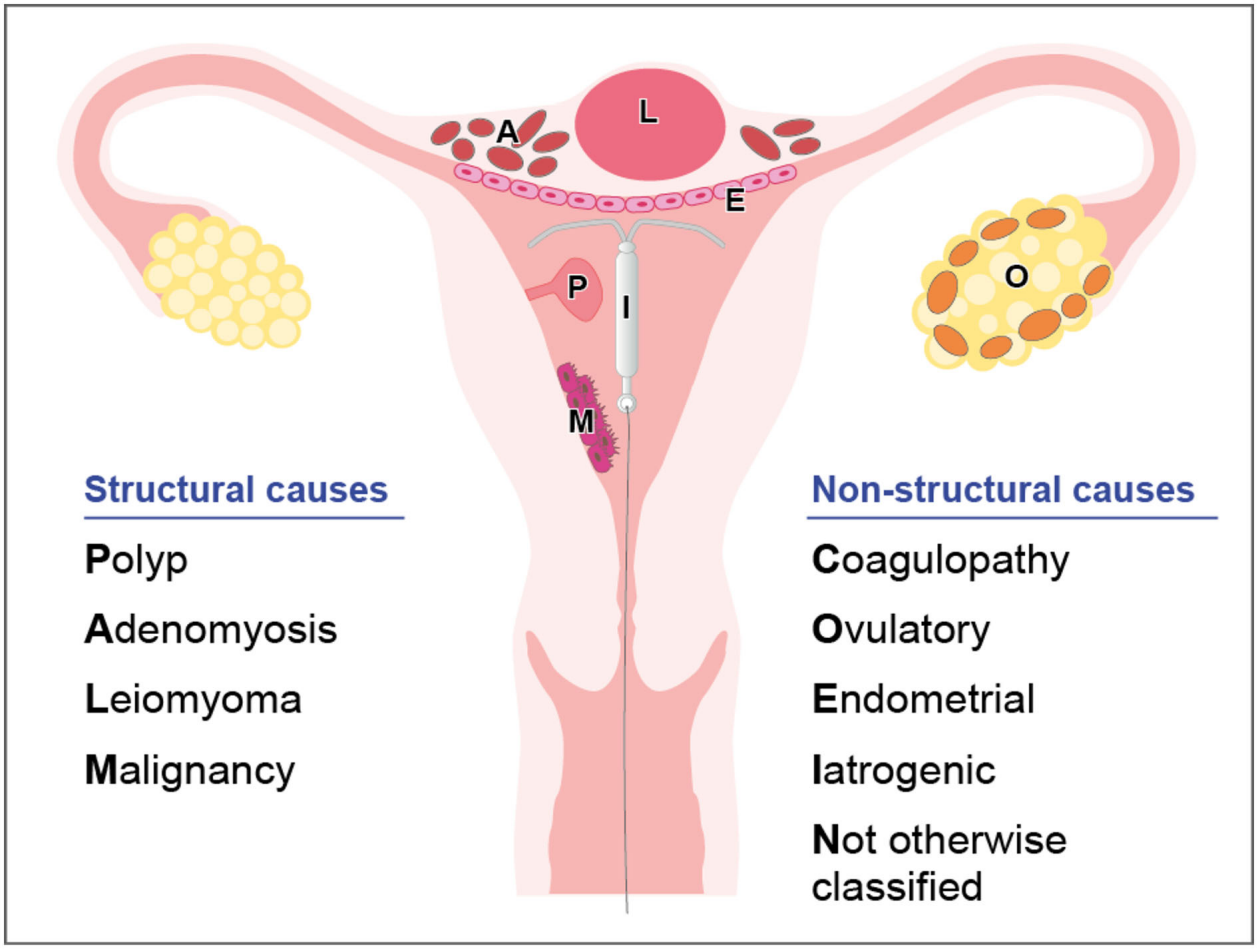
FIGO classification of causes of AUB; “PALM-COEIN.”

There are nine main categories: **P**olyp, **A**denomyosis, **L**eiomyoma, **M**alignancy and **H**yperplasia, **C**oagulopathy, **O**vulatory dysfunction, **E**ndometrial, **I**atrogenic, and **N**ot otherwise classified. In general, the components of the PALM group are discrete (structural) entities that may be identified visually with imaging techniques and or histopathology, whereas the COEIN group is related to entities that are not defined by imaging or histopathology (nonstructural) (Figure 1) [2].

The term “DUB,” which was previously used as a diagnosis when there was no systemic or locally definable structural cause for AUB, should be abandoned [8]. These women generally have one or a combination of coagulopathy, a disorder of ovulation, or primary endometrial disorder—the last of which is most often a primary or secondary disturbance in local endometrial hemostasis [2]. Similarly, the terms menorrhagia and metrorrhagia should also be abandoned [3].

### Demographics

There is a significant clinical burden associated with AUB, affecting 14–25% of women in the reproductive age group [14, 15]. About 20% of the 1.2 million referrals to specialist gynecologist services concern women with HMB [15]. Current NICE guidance clearly states that HMB should be managed in the context of improving the woman's quality of life, rather than treating a target blood loss volume [4]. A recent HMB audit by the Royal College of Obstetricians and Gynaecologists, assessing patient outcomes and experiences in England and Wales, reported that 1-year post referral only 30% of women (including those managed with surgery) were “satisfied” (or better) at the prospect of current menstrual symptoms continuing, as currently experienced, for the next 5 years [15].

Thus, menstrual problems represent a clinical area of unmet need. Poor satisfaction with standard treatment options often results in women opting for major surgery such as hysterectomy. Such women would benefit from a tailored approach, both for diagnosis and treatment, highlighting the deficiency of biomarkers in this area.

Advances in genomics have ushered in a new era called “personalized” or “precision” medicine, which takes into account individual genetic and other sources of variability in disease treatment and prevention. A strong rationale for the shift toward precision medicine was laid by the National Research Council [16]. Genomic profiling of endometrium may offer a significant step forward, in the healthcare for women with AUB/HMB, both by directing patients to most suitable current treatments and by informing new avenues for effective and personalized medical management.

In keeping with the PALM-COEIN classification system, AUB-E possibly represents a primary endometrial disorder, while AUB-L and AUB-A, which are still poorly understood, potentially, may represent, a secondary endometrial disorder.

### Polyps and AUB (AUB-P)

Endometrial polyps are epithelial proliferations arising from the endometrial stroma and glands. The majority are asymptomatic The reported prevalence of endometrial polyps varies widely and ranges from 7.8 to 34.9%, depending on the definition of a polyp, the diagnostic method used, and the population studied [17–20]. There are no identified biomarkers for polyps in clinical use, and their diagnosis relies on imaging, ultrasonography (USS), sonohysterography, and hysteroscopy [4].

### Adenomyosis and AUB (AUB-A)

Adenomyosis is defined as the presence of ectopic endometrial glands and stroma in the myometrium, although it remains a poorly understood entity. The prevalence of adenomyosis is difficult to ascertain because of a wide variation in diagnostic criteria both with imaging modalities and with histology. It has been estimated that histological confirmation of adenomyosis ranges from 5 to 70% of patients who undergo hysterectomy [21]. Adenomyosis is thought to cause HMB, dysmenorrhea and infertility [22] although not all studies agree that HMB is a causal association [23].

Risk factors for adenomyosis include increasing parity, termination of pregnancy, uterine curettage, and caesarean birth, all of which may disrupt the endo-myometrial junction and thereby allow infolding of the endometrium with direct myometrial invasion [24, 25]. There also appears to be an association between estrogen exposure and development of adenomyosis. Increasing age with increased duration of estrogen exposure, and tamoxifen use positively correlate with adenomyosis risk [26]; interestingly, cigarette smoking may be protective [27]. Increasing age with cellular damage and repair may be contributory. Other gynecological conditions that may be associated with adenomyosis include uterine fibroids and endometriosis, although there is a debate if the latter is a separate entity at all [28]. At a cellular level, matrix metalloproteinases (MMPs) may initiate damage to the endo-myometrial junction via the basement membrane [29], and cyclo-oxygenase (COX) enzymes, vascular endothelial growth factor (VEGF), and stem cell progenitors may contribute to the development of adenomyosis [30, 31].

In a study by Li et al, aberrations in angiogenesis were proposed as mechanisms to explain histological changes in adenomyosis. A positive correlation was observed between VEGF expression and MMP-2 and MMP-9 expression. A positive correlation was also found between mean vessel diameter (MVD) and MMP-2 or MMP-9 expression. It seems, therefore, that the elevation of MMP-2 or -9 expression may represent an important factor in the development of the disease, contributing to the invasion of endometrial tissues into the myometrium and angiogenesis in adenomyotic implants [32].

Recent evidence also points to epithelial to mesenchymal transition (EMT) in the pathogenesis. Epithelial-to-mesenchymal transition is a process characterized by a loss of polarity of epithelial cells and transition to a mesenchymal phenotype, which at a molecular level involves downregulation of epithelial markers (e.g. E-cadherin) and overexpression of mesenchymal markers (e.g. fibronectin and vimentin), and activation of a number of transcription factors, including Snail, Slug, Twist, Zeb1, and SIP1 [33].

Reduced apoptosis and increased proliferation of the eutopic endometrium could play a role in the pathophysiology of adenomyosis [34]. This was further quantified using a Ki-67 (a nuclear and nucleolar protein that is strictly associated with cell proliferation) labeling index by Yang and colleagues [35]. The study involved analyzing eutopic endometrium in premenopausal women with and without adenomyosis who underwent a hysterectomy. The endometrium was separated into endometrial stromal cells (ESCs). Markers for proliferation were investigated using nonradioactive assay kits, and immunohistochemistry (IHC) and those for apoptosis was analyzed using fluorescence-activated cell sorter. Sotnikova et al have suggested impaired cytokine production in the development of adenomyosis [36].

Studies also propose an increased synthesis of local estrogen and possibly, progesterone resistance in women with adenomyosis. Increased aromatase and estrone sulfatase activity in glandular cells of eutopic and ectopic endometrial tissues in women with adenomyosis can contribute to increased local uterine/endometrial estrogen production, and reduced progesterone receptor B isoform (PR-B receptors) may contribute to local progesterone resistance [37, 38].

The concept of repeated tissue injury and repair in women with adenomyosis is supported in a study by Liu and colleagues. They propose that platelet-induced activation of the TGF-β/Smad signaling pathway may be a driving force in EMT, fibroblast-to-myofibroblast transdifferentiation, and smooth muscle metaplasia in the development of adenomyosis leading to fibrosis. Platelets may also be involved in uterine hyperactivity and myometrial hyperinnervation, potentially contributing to adenomyosis symptoms [39].

Diagnosis of adenomyosis is based on histology (at hysterectomy) and imaging. The common imaging modalities used for nonhistological diagnosis of adenomyosis include transvaginal ultrasound (TVUS) and magnetic resonance imaging (MRI), although a clear consensus on imaging criteria remains lacking. A recent systematic review confirms that TVUS 2D is effective and should be considered as the first-line ultrasound imaging method for the diagnosis of adenomyosis. Enhancing tools such as TVUS 3D improved upon sensitivity when used with poor definition of the junctional zone, while no improvement was noted in the overall sensitivity compared to TVUS 2D [40]. Newer imaging techniques such as elastography in addition to conventional ultrasound may hold potential in the future to assist with diagnosis of uterine focal lesions and may be useful in preoperative planning [41]. A recent study has also proposed that TV elastography can improve the diagnostic accuracy for adenomyosis, especially in differentiating it from uterine fibroids. The study also suggests a role for elastography in the assessment of the developmental stage of adenomyotic lesions and a guide for the best treatment modality for the patient [42].

A meta-analysis comparing TV US and MRI in the adenomyosis concluded that both techniques showed high levels of accuracy, although a correct diagnosis was obtained more often with MRI [43]. The Morphological Uterus Sonographic Assessment (MUSA) group has proposed uniform criteria for the diagnosis of both uterine fibroids and adenomyosis to facilitate consistent reporting in both daily clinical practice and for research purposes [44, 45].

Several biomarkers have been explored in research settings for diagnosing adenomyosis, but none have been adapted for clinical use. Caveolin (CAV) proteins are the fundamental components of caveolae that form different structural and functional microdomains in a wide variety of cell types. A study evaluated the expression of CAV 1 in the ESCs in the human uterus affected by adenomyosis and concluded that loss of stromal CAV1 expression may play a critical role in the pathogenesis of adenomyosis. Loss of stromal CAV1 expression enhanced metastasis of ESCs and enabled increased growth, migration, and invasion of endometrial epithelial cells (EECs) that might involve the release of RANTES in the stroma of the ectopic lesion. RANTES (also termed CCL5), a chemokine for monocytes and activated T cells, significantly correlates with the severity of stages and dysmenorrhea in women with deep infiltrating endometriosis. The expression level of RANTES in the ectopic ESCs of adenomyosis patients was significantly higher than that of the eutopic counterpart. Silencing of stromal CAV1 in ESCs could trigger nitric oxide (NO) and prostaglandin E2 (PGE2) production in ESCs, potentially contributing to the symptom of dysmenorrhea [46].

Moesin, a protein encoded in human by the MSN gene, has been proposed as a biomarker for adenomyosis. Using proteomic analysis, a higher expression of moesin was noted in adenomyosis versus normal endometrium. This was initially identified using IHC with a higher expression in ESC than in EEC and confirmed using RT-PCR and western blot. An association between moesin as a marker for EMT has been already proposed and may contribute to our understanding of the pathophysiology of adenomyosis. Adenomyosis development mimics the process of tumor metastasis, which is characterized by progressive transmyometrial invasion of endometrial cells and neovascularization in ectopic lesions. To explain the invasiveness seen in adenomyosis, the authors propose a further review of the phosphorylation of moesin in women with adenomyosis, as in certain tumors such as invasive gastric adenocarcinoma, the extent of invasiveness correlates with moesin expression [47].

A common method employed for biomarker discovery is proteomics, which is, in essence, a large-scale study of proteins. Proteomic-based approaches for biomarker investigation can be employed in different aspects of medicine, such as elucidation of pathways affected in disease, identification of individuals who are at a high risk of developing disease for prognosis and prediction of response, identification of individuals who are most likely to respond to specific therapeutic interventions, and prediction of which patients will develop specific side effects. Proteomics analysis has been used to compare the differential protein expression profile between matched ectopic and eutopic endometrium of adenomyosis. The study showed that a group of estrogen-responsive proteins were significantly altered and amongst them, Annexin 2 (ANXA2) was identified as a key player in adenomyosis development by inducing both metastasis and proangiogenesis of adenomyotic endometrial cells. The invasive and metastatic potential involved in adenomyosis was achieved by ANXA2-induced β-catenin/T-cell factor associated EMT-like switch in endometrial cells, and the proangiogenic capacity in local lesion was enhanced via ANXA2/HIF-1α/VEGF-A pathway activation [48].

Proteomic analysis using matrix-assisted laser desorption ionization time-of-flight mass spectrometry (MALDI-TOF-MS) has been studied in women with adenomyosis and endometriosis. The study compared protein peaks using the MALDI-TOF-MS system in the serum of women with endometriosis or adenomyosis to controls and identified a possible biomarker for the conditions, but was unable to differentiate between endometriosis and adenomyosis [49].

The nuclear factor kappa light chain enhancer of activated B cells (NF-κB) pathway has long been considered a proinflammatory signaling pathway, largely based on the role of NF-κB in the expression of proinflammatory genes including cytokines, chemokines, and adhesion molecules. It also plays an important role in apoptosis and cellular growth [50]. NF-κB activity is regulated by a family of proteins known as IκBs. There are two pathways of (NF-kB) activation that are known currently. The canonical pathway is triggered by microbial products and proinflammatory cytokines such as TNFα and IL-1, while the alternative pathway is triggered by TNF-family cytokines but not TNFα [51, 52]. Immunoreactive proteins have been studied as potential biomarkers, including p65, p50, and p52. Nuclear p65 immunoreactivity was positively associated with heavier menses and decreased PR-B, and increased nuclear p65 immunoreactivity in the ectopic endometrium was associated with the severity of dysmenorrhea in women with adenomyosis [34, 38].

Huang et al. proposed that an imbalance between apoptosis and proliferation may contribute to the pathogenesis and progression of adenomyosis. The presence of ectopic endometrium in adenomyosis is likened to a tumor-like invasiveness, although poorly understood [53]. Tyrosine kinase receptor B (TrkB) is a neurotrophic receptor and contributes to tumor cells’ resistance to apoptosis, and the acquisition of invasive and metastatic abilities [54]. Moreover, overexpression of TrkB in several types of human malignancy supports this hypothesis. The study concluded that TrkB protein and TrkB mRNA in adenomyotic endometrium were elevated and positively correlated with the degree of dysmenorrhea. This may contribute to our understanding of the pathogenesis of adenomyosis and may represent a potential biomarker for disease progression in the future [53].

Tissue factor (TF) is a cell membrane-bound glycoprotein and a member of the cytokine receptor family [55]. Tissue factor is mainly expressed in ESC and is regulated by progesterone. Tissue factor is involved in the pathogenesis of endometriosis, possibly in angiogenic and inflammatory signaling and has been evaluated as a biomarker in women with endometriosis [56]. Tissue factor elevation in women with endometriosis is thought to explain symptoms of dysmenorrhea and HMB, and given the similarities between endometriosis and adenomyosis, the authors proposed to identify elevated TF expression in women with adenomyosis [57]. The study showed increased TF immunoreactivity in the adenomyotic endometrium (eutopic and ectopic) versus controls (no adenomyosis) and had a strong association with HMB and dysmenorrhea [57].

SLIT is a secretory glycoprotein that acts via its receptor ROBO, a transmembrane protein. SLIT-ROBO system is reported to function as a chemoattractant to recruit vascular endothelial cells to sites for vasculogenesis [58]. Increased SLIT expression correlates with increased MVD and is a marker for tumor angiogenesis [59]. SLIT immunoreactivity is increased in endometriosis. Its elevation may be a constitutive biomarker for recurrence of endometriosis, and given the similarities between adenomyosis and endometriosis, SLIT has been explored as a potential biomarker in women with adenomyosis [60]. In comparison to the normal endometrium, Nie and colleagues demonstrated that SLIT expression was higher in the ectopic endometrium of women with adenomyosis, while ROBO1 immunoreactivity and MVD were higher in both eutopic and ectopic endometria of women with adenomyosis and that these biomarkers positively correlated with the severity of dysmenorrhea [61]. Table 1 summarizes the researched biomarkers and potential pathogenetic mechanisms of adenomyosis.

**Table 1.** Researched biomarkers and potential pathogenesis in adenomyosis.

• Increased MMP expression [32]
• Epithelial to mesenchymal transition (EMT) [33]
• Increased proliferation (KI-67) and reduced apoptosis in the eutopic endometrium [35]
• Increased local estrogen production—increased aromatase and estrone sulfatase activity [35]
• Increased local progesterone resistance mediated by progesterone receptor B isoform (PR-B receptors) [38]
• Repeated tissue injury and repair—platelet-induced activation of the TGF-β/Smad signaling pathway [39]
• Reduced expression caveolin (CAV) proteins [44]
• Increased moesin expression [47]
• Increased Annexin 2 (ANXA2) expression [48]
• Activation of NF-κB [51, 52]
• Increased expression of TrkB [51,52]
• Increased SLIT/ROBO expression [60, 61]
• Imaging—elastography [41, 42]

### Leiomyomas and AUB (AUB-L)

Uterine fibroids (myomas, leiomyomas) are the most common benign tumors in women of reproductive age present in almost 80% of all women by the age of 50 [62]. Fibroids tend to be twice or even three times more common in black women as compared to other racial or ethnic groups [63]. The association between AUB and fibroids is complex and poorly understood, as women with fibroids may be asymptomatic; however, a strong association exists between submucous myomas and AUB, demonstrated as early as 1956 [64].

The proposed mechanisms of how fibroids may cause AUB include an increase in the endometrial surface, an increase in uterine vascularization, changes in patterns of myometrial contractility, ulceration of the surface of a myoma, myoma degeneration, and uterine venous ectasia by compression effect from the myoma(s) [65]. These proposed mechanisms often relate to fibroid size, but cannot explain completely the relationship between AUB and fibroids. There is a correlation between AUB and the degree of distortion and penetration of the uterine cavity associated with the fibroid(s). Submucous myomas (FIGO 0, 1, 2, and 3) are thought to be most symptomatic [66]. Distortion of the uterine cavity by fibroids is also proposed to explain other symptoms such as infertility [67].

In recent years, our understanding of fibroids at a molecular level and cellular has significantly improved. Although several potential biomarkers have been identified (discussed below), none are in clinical use. It is well established that fibroids are monoclonal tumors arising from the smooth muscle cells of the myometrium. Fibroids contain three different cell populations: fully differentiated smooth muscle cells, intermediate cells, and fibroid stem cells, which in turn are crucial to fibroid growth. A genetic hit in the myometrial stem cell can produce fibroid stem cells. These genetic hits include mutations in the mediator complex (MED) 12 gene and chromosomal rearrangements on the high mobility group A (HMG2A) gene on the long arm of chromosome 12 [68–71].

Endocrine-disrupting chemicals (EDCs) are substances in our environment, food, and consumer products that interfere with hormone biosynthesis, metabolism, or action resulting in a deviation from normal homeostatic control or reproduction and there is evidence to suggest that exposure to EDCs, especially in critical phases of uterine development such as in utero and early childhood, may result in genetic mutations influencing fibroid growth [72, 73].

Fibroids are steroid hormone-dependent tumors; however, unlike differentiated fibroid cells, fibroid stem cells have a very low expression of estrogen and progesterone receptors, indicating that these hormones exert their tropic effects on fibroid stem cells via a paracrine mechanism. Fibroids also secrete increased transforming growth factor-beta 3 (TGF-β3) in response to steroids (see below). TGF-β3 is a cytokine that is involved in cell differentiation, embryogenesis, and development and is believed to regulate molecules involved in cellular adhesion and extracellular matrix (ECM) formation. TGF-β has a direct effect on fibroid ECM production, stimulating collagen expression as well as plasminogen activator inhibitor-1 expression [74]. The role of TGF-β in fibrotic processes such as liver cirrhosis and pulmonary fibrosis is well established, and further research may improve our understating of fibrotic pathways associated with fibroids [75].

The role of the WNT/β-catenin pathway is also of importance in fibroid growth. Mutations in MED 12 genes are believed to lead to alterations in the WNT/B-catenin pathway expression and signaling. This results in degradation of cytoplasmic β-catenin and increased nuclear β-catenin, which is associated with increased fibroid burden in murine models [76, 77]. In human fibroid cells, silencing the MED 12 gene results in decreased WNT/β-catenin pathway signaling, thereby slowing fibroid growth [70]. The WNT/β-catenin pathway also results in increased expression of TGF-β3. A recent study by Sinclair et al. suggested that leiomyoma-secreted TGF-β3 induces BMP-2 resistance in the endometrium by downregulation of BMPR-2, likely causing defective endometrial decidualization. TGF-β3 also reduces expression of plasminogen activator inhibitor-1 (PAI-1), ATIII, and thrombomodulin in the endometrium, likely contributing to AUB/HMB. In the past, TGF-β3 has been shown to be involved in ECM remodeling and proliferation which could modulate fibroid growth [78].

AUB/HMB associated with fibroids may be explained by a complex interplay between coagulation, neo-angiogenesis, and vasoconstriction. As described above, fibroids secrete TGF-β3, which in turn may alter normal hemostatic and fibrinolytic pathways through PAI-1, ATIII, and thrombomodulin in endometrium [78]. Evidence also suggests an increased expression of fibroblast growth factor and fibroblast growth factor receptor in the endometrium of women with fibroids [79]. Other angiogenic factors such as heparin-binding epidermal growth factor, platelet-derived growth factor, VEGF, parathyroid hormone-related protein, and prolactin are also altered in women with fibroids [80]. This could explain altered neo-angiogenesis and HMB with fibroids. Endothelin-1 (ET) and prostaglandin F2 alpha (PGF_2_α) are potent vasoconstrictors that regulate menstruation [81]. Vasoconstrictors that regulate myometrial contractility (ET-1, PGF_2_α) and spiral arteriole vasoconstriction (ET-1) are altered in women with fibroids [82, 83]. PGF_2_α production is increased in women with uterine fibroids [82]. Endothelin-1 acts via the ET_A_-R and ET_B_-R receptors and higher levels of endothelin-1 have identified in the endometrium as compared to the fibroid tissue and myometrium. In addition, higher levels of ET_A_-R are identified in the myometrium compared to fibroid tissue and vice versa for ET_B_-R. These alterations suggest disordered ET function in women with uterine fibroids [83]. The consequence of perturbed expression of these vasoconstrictors results in alerted myometrial contractility and dilatation of endometrial stromal venous spaces and may explain HMB associated with fibroids.

Changes in circulating levels of cytokines such as interleukins (IL)-10, 13, and 17 have been identified in women with fibroids [84]. In general, an infection is accompanied by an inflammatory process; however, an inflammatory response evidenced by altered cytokine levels in the endometrium (out with infection), as a mechanism for HMB associated with fibroids, remains to be established.

A clinical and functional genomics analysis in women with fibroids was undertaken and concluded that that intramural leiomyomas not affecting the endometrial cavity alters the expression pattern of some endometrial genes, but the genes involved in implantation are not affected. The study identified that the expression of 69 genes strongly correlated with the size of the myoma, and 26 genes did so positively, whereas 43 did so negatively. Among the genes that were upregulated with the size of the intramural fibroid, there was an association with larger blood vessel size, a feature that correlates well with the angiogenesis involved in fibroid vascular supply and growth. Similarly, immune response and response to wounding were underrepresented when a fibroid was present. An impairment of maturation and differentiation of lymphocytes in women with large leiomyomas suggested a decrease in the local immune response [85].

Despite all the advances discussed above, a biomarker for identifying causal factors underlying AUB in women with fibroids remains elusive currently, and reliance for identifying the presence of uterine fibroids is placed on imaging modalities (USS, MRI, sonohysterography) and hysteroscopy. Table 2 summarizes the researched biomarkers and potential pathogenetic mechanisms of leiomyomas.

**Table 2.** Researched biomarkers and potential pathogenesis—fibroids.

• Chromosomal rearrangements on the high mobility group A (HMG2A) gene [71]
• Mutations in the mediator complex (MED) 12 gene [68–70]
• Exposure to endocrine-disrupting chemicals (EDCs) [72, 73]
• Increased production of transforming growth factor-beta 3 (TGF-β3) with increased BMP-2 resistance [74]
• WNT/β-catenin pathway [70, 76–78]
• Altered cytokines such as interleukins (IL) -10,13,17 [84]
• Imaging—elastography [41]

### Malignancy and AUB (AUB-M)

It is beyond the scope of this article to discuss malignancy-related biomarkers as it is primarily focused on benign pathology.

### Coagulopathy and AUB (AUB-C)

Coagulopathies are reported to affect 13% of the women presenting with HMB [86]. The systemic disorders of hemostasis may be identified in 90% of women using a structured history (see Table 3) [87].

**Table 3.** Detection of coagulopathies (adapted from Kouides et al [87]).

Structured history—positive screen if
a. Excessive menstrual bleeding since menarche, or
b. History of one of the following—postpartum hemorrhage, surgery-related bleeding, or bleeding associated with dental work, or
c. History of two or more of the following—bruising greater than 5 cm once or twice/month, epistaxis once or twice/month, frequent gum bleeding, family history of bleeding symptoms.

There are clearly defined biomarkers for this cause of AUB, so long as health professionals are meticulous at screening women at risk and offering onward referral to appropriate specialists. These biomarkers include a full blood count, measurement of individual coagulation factor quantity and/or activity, D-Dimer, fibrinogen, international normalized ratio, partial thromboplastin time, prothrombin time, thrombin time, platelet function test, ristocetin cofactor, von Willebrand factor antigen, and several more. It is beyond the scope of this article to discuss individual biomarkers for AUB-C.

In the original FIGO PALM-COEIN system, women with AUB associated with the use of anticoagulants were categorized with coagulopathies (AUB-C). In the updated classification (2018), they are considered iatrogenic and classified as AUB-I. This includes the modern, nonvitamin-K antagonists such as rivaroxaban that appears to have a greater impact on the volume of menstrual bleeding than the traditional, vitamin K antagonists such as warfarin [13, 88].

### Ovulatory disorders and AUB (AUB-O)

Anovulation is observed at extremes of age, in association with endocrine disorders such as hypothyroidism, polycystic ovarian syndrome, hyperprolactinemia, and with factors such as mental stress, extremes of weight, excess exercise, and even drugs that interfere with the hypothalamic–pituitary–ovarian axis such as dopamine agonists. Anovulatory cycles tend to present as an alteration in cycle length (often > 38 days) and AUB/HMB due to the effect of unopposed estrogen on the endometrium.

Although a well-structured history and examination may identify many cases, specific tests may be ordered to rule out endocrinopathies. Thus, there are clinically relevant biomarkers for this cause of AUB, such as serum thyroid stimulating hormone and thyroxine levels, prolactin levels, gonadotropin levels (FSH/LH), sex hormone binding globulin, free androgen index, and so on. It is beyond the scope of this article to discuss individual biomarkers for endocrinopathies contributing to AUB-O.

Bao et al. have presented data to identify serum amyloid protein A (SAA) as a potential biomarker to differentiate between ovulatory and anovulatory AUB. Using surface-enhanced laser desorption ionization TOF-MS, they identified three protein peaks corresponding to SAA, VEGF, and anti-vitamin K epoxide reductase (VKOR). Given that SAA is highly expressed in individuals with nonimmune inflammation, the authors hypothesized a similar response in women with AUB, that SAA was highly expressed in the menses (sera and supernates) of anovulatory and ovulatory women with AUB versus controls. The role of VEGF is well established in menstruation and endometrial repair [89]. In this study, VEGF was highly expressed in ovulatory women with AUB but poorly expressed in anovulatory patients suggesting a possible aberration in angiogenesis in anovulatory AUB. Vitamin K is essential in the clotting cascade and requires VKOR for this process. The authors noted a poor expression of VKOR in the menses of women with ovulatory AUB possibly suggesting a defect in clotting [90].

### Endometrial disorders and AUB (AUB-E)

AUB that occurs in the absence of an identifiable histological or structural cause (AUB-L, AUB-A, AUB-P), in the context of regular menstrual cycles (ovulatory) and with coagulopathy ruled out, in the absence of iatrogenic causes (AUB-I) usually represents a primary endometrial disorder (AUB-E). AUB-E is thought to be caused by a local disturbance(s) in endometrial function—deficiencies or excesses of proteins or other entities that have an adverse impact on hemostasis, normal angiogenesis, vascular integrity, or endometrial repair. AUB-E is a diagnosis of exclusion; a well-structured history and examination often help, but there is no commercially available testing. Hence, a clear role for developing biomarkers exists for this cause of AUB.

The endometrium is a complex multicellular tissue that lines the inside of the endometrial cavity and involves interactions of immune, endocrine, and vascular systems. It is morphologically divided into functional and basal layers. The functional layer occupies the upper two-thirds of the endometrium. During endometrial repair and proliferation, mitosis occurs in the functional layer of the endometrium, a highly active layer consisting of glands supported by stroma. Studies now demonstrate that the basal layer may not serve as a source of stem cells for endometrial regeneration after normal menstruation. Instead, changes in the microenvironment may reprogram the few functional cells remaining after menstruation to regenerate a new functional layer [91].

It is well established that progesterone withdrawal secondary to the demise of the corpus luteum in the absence of pregnancy is the signaling event for the onset of menstruation. A key role is played by decidualized ESCs, as they remain responsive to progesterone through the secretory phase. They retain progesterone receptor (PR) expression, thereby allowing the endometrium to respond to progesterone withdrawal. Progesterone withdrawal is proposed to have two major effects: (a) increased levels of cytokines and prostaglandins into the endometrium and consequently (b) influx of leukocytes, activation of MMPs, and destruction of the ECM [92]. The action of MMPs is thought to be independent to progesterone withdrawal after an initial inflammatory response.

Neutrophil type leukocytes predominantly increase in the endometrium and contain high levels of MMPs and can activate local MMPs, initiating endometrial breakdown. Increased B-cell lymphoma 2 (BCL 2– an apoptosis regulator) levels secondary to progesterone withdrawal, limit neutrophil activity in the endometrium and prevent a damaging chronic inflammatory response [93]. Macrophages also increase perimenstrually and produce cytokines and proteases and are involved in tissue remodeling and debris removal [94, 95].

Inflammatory responses in the endometrium are mediated via the NF-κB pathway, secondary to steroid hormone withdrawal. NF-kB increases the transcription of a wide variety of genes, including cytokines (IL-1, IL-6), chemokines (CXCL8/IL-8, chemokine ligand 2/CCL-2), and adhesion molecules [96]. Increased IL-8 mRNA expression in premenstrual endometrium and localization to perivascular cells by the withdrawal of progesterone has been shown by Milne et al. [97]. A role for cyclo-oxygenase (COX)-2 following progesterone withdrawal has also been demonstrated [98]. Inhibition of the COX enzymes or NF-kB at the time of progesterone withdrawal significantly decreased the amount of bleeding and endometrial breakdown and leukocyte influx in a murine model [99].

Menstruation has been proposed to occur also as a result of a physiological process of ischemia and reperfusion. Ischemia has not been detected in the human endometrium during menstruation to date; however, evidence supports the occurrence of hypoxia in the endometrium. Markers for hypoxia (CAIX and hypoxia inducible factor-1α [HIF-1α]) have been detected in the human endometrium during menstruation [100]. The current evidence supports the role of hypoxia and HIF-1α in the process of endometrial repair during menstruation [101].

Vasoconstriction of the uterine spiral arterioles mediated by PGF_2α_ and ET-1 is considered to play a role in determining blood loss during menstruation. PGE_2_ is known to have a vasodilatory effect [102]. Evidence supports the theory of HMB secondary to a reduced endometrial expression of ET-1 and an altered PGF_2α/_PGE_2_ ratio. The reduced maturity of the uterine spiral arteriole vessel wall, increased gaps in the endothelial cell lining, and reduced vascular smooth muscle proliferation may all contribute to HMB [103–106].

Cessation of menstruation relies on an intact endometrial coagulation system. Endometrial endothelial injury initiates immediate activation and aggregation of platelets to form a plug. The subsequent stage of hemostasis involves the formation of fibrin via the coagulation cascade. Tissue plasminogen activator and urokinase plasminogen activator drive the production of plasmin, and PAI inhibits fibrinolytic activity [107]. There is evidence that an overactive fibrinolytic system in the endometrium interferes with hemostasis and contributes to HMB [108].

Angiogenesis and spiral arteriole maturation are essential components of repair during menstruation, a process that is usually completed by cycle day 5. Vascular endothelial growth factor, a key mediator of vascular function, is increased in women at menses, and is regulated by hypoxia [109]. This process is independent of steroid hormones. Recently, interest has increased in the role of immune cell influx at the time of menstruation the role they may play in the regulation of endometrial bleeding. The uterine natural killer (uNK) cells may play an important role in spiral arteriole maturation, which in turn will impact upon vasocontraction and potentially reduced menstrual blood loss [110]. Dysregulation of uNK cells in HMB has also been demonstrated, which may have an impact on endometrial vascular development and or endometrial preparation for menstruation. [111].

Newer research has focused on the role of hypoxia and the HIF-1α in endometrial repair using a murine model of simulated menstruation. Validation of the mouse model of menstruation has already been performed [95]. The study by Maybin et al. demonstrated reduced endometrial HIF-1α in women with AUB/HMB. The study suggests that HIF-1α regulates response within cells to low oxygen levels (hypoxia), increasing the production of a number of repair factors and therefore playing an important role in repair of the denuded endometrial surface [101].

Despite the vast improvements in the understanding of the cellular and molecular basis of menstrual physiology, clinically usable biomarkers remain lacking in women with AUB-E.

### Iatrogenic AUB (AUB-I)

AUB may be associated with the use of exogenous steroids, usually as continuous estrogen and or progesterone therapy results in unscheduled bleeding (BTB) [112]. Drugs that interfere with ovarian steroid release may have a similar effect (GnRH agonists and antagonists) and aromatase inhibitors. The use of intrauterine contraceptive devices may contribute to chronic endometritis (CE) and AUB [113]. A structured history and examination and exclusion of other causes help to secure the diagnosis. Often cessation of the drug or removal of the device (implant, intrauterine device) helps resolve the problem. The role of biomarkers in this category is limited.

### Not otherwise classified (AUB-N)

Entities such as CE (not secondary to IUD use), arteriovenous malformations (AVMs) [114], endometrial pseudoaneurysms, and myometrial hypertrophy have been associated with or contribute to AUB/HMB. Cesarean sections scar defects such as “isthmoceles” may also contribute to AUB [115, 116]. In addition, there may be other disorders that would be defined only by biochemical or molecular biology assays that should be placed in this category. Considering these entities are extremely rare, the search for a viable endometrial biomarker is only of academic value.

Most women with AVMs, endometrial pseudoaneurysms, and myometrial hypertrophy will be diagnosed using imaging techniques. Chronic endometritis is poorly understood but plays an important role in AUB and poor reproductive outcomes and is currently diagnosed by histology. A study by Tortorella et al, proposed the use of biomarkers in the menstrual effluent for diagnosing CE. They identified that proinflammatory cytokines are increased in menstrual effluents of women with CE with IL-6 and TNF-alpha having a high screening capacity for the condition [117]. The role of *Chlamydiatrachomatis* and AUB has also been described, and its prevalence is thought to be underestimated as a cause of AUB [118].

### Future directions

Although to date, there is a limited success in the clinical use of biomarkers for women with AUB, this remains an area of unmet need. In a medical context, the word “phenotype” is used to refer to some deviation from normal morphology, physiology, or behavior. Deep phenotyping can be defined as the precise and comprehensive analysis of phenotypic abnormalities in which the individual components of the phenotype are observed and described. The emerging field of precision medicine aims to provide the best available care for each patient based on stratification into disease subclasses with a common biological basis of disease [119]. There is a clear need for developing a system of deep phenotyping for women with AUB, such that individualized and personalized care can be offered to ensure best results with treatment strategies.

A role for the endometrial microbiome has been proposed, and a study by Pelzer et al. using microbial community profiling revealed differences in the endometrial microbial community profiles for (1) the endocervix compared to the endometrium, and (2) women with HMB versus dysmenorrhea [120]. This allows for further exploration in this field to try and understand the pathogenesis and develop management strategies for women with AUB.

The role of exosomes using proteomic analysis in predicting adverse pregnancy outcomes and suggesting pathophysiologic mechanisms has been explored in the context of preterm birth [121]. Exosomes act as proxies for cells and therefore serve as better biomarkers than secreted biochemicals from cells. Exosomes may hold a role in the future as tools for “noninvasive” tissue sampling in women with AUB. Exosomes derived from menstrual blood have already been used for other applications [122]. This remains an unexplored field with the potential to discover new biomarkers. Uterine fluid obtained by lavage or as aspirates, menstrual loss supernatants, and sera may all have a future role to play [90, 123].

Current pathology practice utilizes chromogenic IHC, and improving the technology available may allow us to identify clinically usable biomarkers. Multiplexed IHC (mIHC) approaches are now available, offering greater insights into disease heterogeneity and the characterization of systems biology mechanisms driving disease, as well as helping to conserve limited tissues. Multiplexed IHC offers greater insight into molecular cascades, preserves tissue context, and allows for improved accuracy through the application of image analysis, with the use of landmark markers specifically to indicate tissue architecture [124]. Application of such technology has already been used in the field of cancer. Kim et al. have demonstrated quantitative proteomic profiling of breast cancers using mIHC, so that individualized cancer therapy can be offered [125].

In keeping with advances in technology, elastography (MRI and USS) is being increasingly used in the assessment of women with fibroids and adenomyosis serving as noninvasive imaging biomarkers. More importantly, there are being used to tailor therapy and predict treatment response for allowing individualized care [126]. A recent study showed that fractional change in stiffness value of uterine fibroids measured by magnetic resonance elastography would be related to the treatment outcomes after magnetic resonance guided focused ultrasound [127].

Identifying differential gene expression in women with AUB/HMB, i.e. a gene signature, may advance our understanding of the mechanisms responsible for HMB and allowed tailored treatment strategies. Steps in this direction have already been made [128].

## Conclusion

AUB is a common and frequently debilitating condition for women worldwide. It has clinical implications and a high cost for the healthcare system. Uterine fibroid tumors were estimated to cost the United States $5.9–34.4 billion annually [129]. HMB is a subjective diagnosis affecting women across the globe. The PALM-COEIN acronym maybe be used as a foundation of care; it improves the understanding of the causes of AUB, and in doing so facilitates effective history taking, examination, investigations, and management [2, 13]. A range of medical and surgical management options are available, the choice of which is guided by the underlying cause of AUB alongside the woman's co-morbidities, fertility wishes, and personal preference in women with AUB-L and AUB-A.

The classification system reveals the lack of effective biomarkers, especially for AUB-E. Tailoring treatments to target increased inflammation, vascular dysfunction, and delayed endometrial repair in women with AUB-E should increase compliance, reduce the need for surgery, preserve fertility (if desired), improve outcomes and patient satisfaction.

Although several potential biomarkers have been discussed in this review, the limitations are that many biomarker studies have only a small number of samples, and studies have not been repeated or validated.

Key factors in the development of technologies for personalized medicine are standardization, integration, and harmonization. For example, the tools and processes for data collection and analysis must be standardized across research sites. Research activity at different sites must be integrated to maximize synergies, and scientific research must be integrated with healthcare to ensure effective translation. There must also be harmonization between scientific practices in different research sites, between science and healthcare, and between science, healthcare, and wider society, including the ethical and regulatory frameworks, the prevailing political and cultural ethos, and the expectations of patients/citizens [130].
